# Supplementary material for: Patterns of multimorbidity and demographic profile of latent classes in a Danish population—A register-based study
Source: PLoS One. 2020 Aug 11;15(8):e0237375. doi: 10.1371/journal.pone.0237375 (PMC7418992; doi:10.1371/journal.pone.0237375)
Supplement: S1 Table — (DOCX) [file pone.0237375.s001.docx]

**Table S1: Included diseases in latent class analyses**

| **Disease** | **Inclusion time (in years)** | **ICD-10 codes** | **ATC codes and indication codes** |
| --- | --- | --- | --- |
| Diabetes | 10 | **E10**  **E11** | **A10A (**not A10AE56)  **A10B** (not A10BJ02) |
| Osteoporosis | 10 | **M80**  **M81**  **M82** | **M05BA01**  **M05BA04**  **M05BA06** (only 150 mg tablets):  **M05BA07**  **M05BB01**  **M05BB03**  **M05BX03**  **G03XC01**  **H05AA02**  **H05AA03** |
| Thyroid diseases | 5 | **E00**  **E01**  **E02**  **E03**  **E04**  **E05**  **E06**  **E07** | **H03** |
| Ischemic heart disease and heart failure | 5 | **I20**  **I21**  **I23**  **I24**  **I25**  **I50**  **I11**  **I13** | **CO1A**  **CO1B**  **CO1D**  **CO1E**  **C01AA05**  **C09A** (only with indication code 430) |
| Pulmonary heart disease and diseases of pulmonary circulation | 5 | **I26**  **I27**  **I28** |  |
| Atrial fabrillation and flutter | 10 | **I48** |  |
| Aortic and mitral valve disease | 5 | **I05**  **I06**  **I34**  **I35** |  |
| Atherosclerosis | 10 | **I70** |  |
| Phlebitis and thrombophlebitis | 5 | **I80** |  |
| Hypertensive diseases | 5 | **I10**  **I12**  **I15** | **C02A**  **C02B**  **C02C**  **C02DA**  **C02L**  **C03A**  **C03B**  **C03D**  **C03E**  **C03X**  **C07C**  **C07D**  **C08G**  **C09BA**  **C09DA**  **C09XA52**  **C02DB**  **C02DD**  **C02DG**  **C07**  **C07F**  **C08**  **C09BB**  **C09DB**  **C09** |
| Disorders of lipoprotein metabolism and other lipidaemias | 10 | **E78.0**  **E78.2**  **E78.4**  **E78.5** | **C10** |
| Crohns’s disease and ulcerative colitis | 10 | **K50**  **K51** |  |
| Irritable bowel disease | 10 | **K58** |  |
| Diseases of liver, biliary tract and pancreas | 10 | **K71**  **K72**  **K73**  **K74**  **K75**  **K76**  **K77**  **K86.1**  **K87** |  |
| Stroke and transient cerebral ischemic attacks and related syndromes and vascular syndromes of the brain in cerebrovascular diseases | 10 | **G45**  **G46**  **I60**  **I61**  **I62**  **I63**  **I64**  **I65**  **I66**  **I67**  **I68**  **I69** |  |
| Epilepsy | 5 | **G40**  **G41** | **N03**  **N05BA** (only with indication code 155 or 753)  **N05CD** (only with indication code 155 or 753) |
| Migraine and other headache syndromes | 10 | **G43**  **G44** | **N02C** |
| Dementia | 10 | **F00**  **F01**  **F02**  **F03**  **G30**  **G31.8B**  **G31.8E**  **G31.9**  **G31.0B** | **N06D** (only with indication code 329, 330, or 331) |
| Parkinson’s disease | 10 | **G20**  **G21**  **G22**  **F02.3** | **N04** |
| Sclerosis | 10 | **G35** |  |
| COPD and chronic lower respiratory diseases | 10 | **J40**  **J41**  **J42**  **J43**  **J44**  **J47**  **J96** | **R03AC18**  **R03AC19**  **R03AL02**  **R03AL03**  **R03AL04**  **R03AL05**  **R03AL06** **R03AL09**  **R03BB04**  **R03BB05**  **R03BB06**  **R03BB07**  **R03DX07**  (all ATC-codes only with indication code 379 or 464) |
| Asthma | 10 | **J45** | **R03DC03** (only with indication code 202, 203, or 822) |
| Chronic kidney disease | 10 | **N18** |  |
| Malignant neoplasms of digestive organs | 10 | **C15-C26** |  |
| Malignant neoplasms of respiratory and intrathoracic organs | 10 | **C30-C39** |  |
| Malignant melanoma of skin | 10 | **C43** |  |
| Malignant neoplasm of breast | 10 | **C50** |  |
| Malignant neoplasms of genital organs | 10 | **C51-C58**  **C60-C63** |  |
| Other malignant neoplasms excluding metastases | 10 | **C00-C14**  **C40-C41**  **C45-C49**  **C64-C68**  **C69-C72**  **C73-C75**  **C81-C96** |  |
| Depression | 5 | **F32**  **F33**  **F34.1**  **F06.32** | **N06A** (only with indication code 168) |
| Anxiety | 5 | **F40.1**  **F41.1** | All prescriptions with indication code 163 or 371 |
| Schizophrenia | 10 | **F20**  **F21**  **F22**  **F25**  **F28**  **F29** | **N05AX13 N05AX12 N05AH03 N05AX08** |
| Bipolar affective disorder | 10 | **F30**  **F31** | **N05A** (only with indication code 461 or 631)  **N06A** (only with indication code 461 or 631) |
| PTSD | 5 | **F43.1** |  |
| Obsessive-compulsive disorder | 10 | **F42** | **N06A** (only with indication code 472 or 596) |
| Eating disorders | 10 | **F50** |  |
| Alcohol attributable diseases | 10 | **G31.2**  **G62.1**  **G72.1**  **K29.2**  **K70**  **K86.0** |  |
| Respiratory allergy | 10 | **J30.1**  **J30.2**  **J30.3**  **J30.4** | **V01AA02**  **V01AA03**  **V01AA05**  **V01AA11**  **R01AC**  **R01AD**  **R06A**  **S01G**  **R01BA52** |
| HIV/AIDS | 10 | **B20**  **B21**  **B22**  **B23**  **B24** |  |
| Inflammatory polyarthropathies | 10 | **M05**  **M06.0**  **M06.8**  **M07.0**  **M07.1**  **M07.3**  **M10.0**  **M10.9** | All prescriptions with indication code 147, 402 or 461 |
| Arthrosis | 10 | **M15**  **M16**  **M17**  **M18**  **M19** |  |
| Spondylopathies and other dorsopathies | 10 | **M40**  **M41**  **M42**  **M43**  **M45**  **M46**  **M47**  **M48**  **M49**  **M50**  **M51**  **M53**  **M54** |  |
| Fibromyalgia | 10 | **M79.7** |  |
| Injuries of nerves and spinal cord and paralytic syndromes | 10 | **G81**  **G82**  **G83**  **S14**  **S24**  **S34**  **T09.3** |  |
| Blindness | 10 | **H54** |  |
| Tinnitus | 10 | **H93.1** |  |
| Congenital malformations, deformations and chromosomal abnormalities | 10 | **Q00-Q99** |  |
