# Supplementary material for: Patterns of multimorbidity and demographic profile of latent classes in a Danish population—A register-based study
Source: PLoS One. 2020 Aug 11;15(8):e0237375. doi: 10.1371/journal.pone.0237375 (PMC7418992; doi:10.1371/journal.pone.0237375)
Supplement: S2 Table — (DOCX) [file pone.0237375.s002.docx]

**Table S2: Disease prevalence in classes in the age group 65+ years**

|  | **’No or few diseases’**  **%** | **’Diabetes, cholesterol’**  **%** | **’Heart diseases’**  **%** | **’Back disease, asthma allergy’**  **%** | **’Many diseases’**  **%** | **’COPD, cancer, liver disease’**  **%** | **’Mental illness, epilepsy’**  **%** |
| --- | --- | --- | --- | --- | --- | --- | --- |
| Diabetes | <1 | 33 | 10 | 4 | 43 | 5 | 11 |
| Osteoporosis | 9 | 8 | 12 | 34 | 28 | 53 | 24 |
| Thyroid diseases | 5 | 8 | 11 | 14 | 16 | 20 | 13 |
| Ischemic heart disease and heart failure | <1 | 18 | 61 | 5 | 67 | 6 | 8 |
| Pulmonary heart disease and diseases of pulmonary circulation | <1 | <1 | 3 | <1 | 5 | 7 | 1 |
| Atrial fabrillation and flutter | <1 | 1 | 84 | 4 | 38 | 4 | 7 |
| Aortic and mitral valve disease | <1 | 3 | 16 | 4 | 14 | 3 | 2 |
| Atherosclerosis | <1 | 3 | 2 | 2 | 19 | 3 | 2 |
| Phlebitis and thrombophlebitis | <1 | <1 | <1 | <1 | 1 | 1 | <1 |
| Hypertensive diseases | 32 | 92 | 96 | 63 | 97 | 54 | 55 |
| Disorders of lipoprotein metabolism and other lipidaemias | 6 | 93 | 42 | 29 | 86 | 14 | 35 |
| Crohns’s disease and ulcerative colitis | <1 | <1 | 1 | 2 | 3 | 4 | 2 |
| Irritable bowel disease | <1 | <1 | <1 | 3 | 3 | 4 | 1 |
| Diseases of liver, biliary tract and pancreas | <1 | <1 | <1 | 1 | 6 | 11 | 2 |
| Stroke and transient cerebral ischemic attacks and related syndromes and vascular syndromes of the brain in cerebrovascular diseases | <1 | 16 | 16 | 11 | 38 | 7 | 30 |
| Epilepsy | <1 | 2 | 1 | 18 | 28 | 4 | 34 |
| Migraine and other headache syndromes | 1 | <1 | <1 | 8 | 5 | 2 | 2 |
| Dementia | 1 | 1 | 4 | 1 | 7 | 2 | 33 |
| Parkinson’s disease | 1 | 1 | 1 | 5 | 7 | <1 | 15 |
| Sclerosis | <1 | <1 | 0 | 1 | <1 | <1 | 1 |
| COPD and chronic lower respiratory diseases | 3 | 8 | 15 | 1 | 41 | 60 | 17 |
| Asthma | 3 | 3 | 3 | 18 | 3 | <1 | 2 |
| Chronic kidney disease | <1 | 2 | 4 | <1 | 15 | 5 | 2 |
| Malignant neoplasms of digestive organs | 2 | 2 | 5 | 3 | 6 | 10 | 3 |
| Malignant neoplasms of respiratory and intrathoracic organs | 2 | 2 | 5 | 2 | 5 | 9 | 3 |
| Malignant melanoma of skin | <1 | <1 | <1 | <1 | 3 | 10 | <1 |
| Malignant neoplasm of breast | <1 | <1 | 1 | <1 | <1 | 1 | <1 |
| Malignant neoplasms of genital organs | 2 | 2 | 2 | 6 | 4 | 12 | 3 |
| Other malignant neoplasms excluding metastases | 3 | 4 | 6 | 3 | 4 | 3 | 3 |
| Depression | 3 | 5 | 5 | 12 | 35 | 13 | 73 |
| Anxiety | <1 | <1 | <1 | 2 | 6 | 2 | 19 |
| Schizophrenia | <1 | <1 | <1 | <1 | 2 | <1 | 21 |
| Bipolar affective disorder | <1 | <1 | <1 | <1 | 1 | <1 | 11 |
| PTSD | <1 | <1 | <1 | <1 | <1 | <1 | <1 |
| Obsessive-compulsive disorder | <1 | <1 | <1 | <1 | <1 | <1 | <1 |
| Eating disorders | <1 | <1 | <1 | <1 | <1 | <1 | <1 |
| Alcohol attributable diseases | <1 | <1 | <1 | <1 | 3 | 4 | 1 |
| Respiratory allergy | 7 | 9 | 8 | 30 | 21 | 18 | 9 |
| HIV/AIDS | <1 | <1 | <1 | 0 | <1 | <1 | <1 |
| Inflammatory polyarthropathies | 1 | 1 | 4 | 10 | 12 | 10 | 1 |
| Arthrosis | 12 | 16 | 20 | 63 | 38 | 19 | 16 |
| Spondylopathies and other dorsopathies | 4 | 6 | 6 | 57 | 41 | 16 | 13 |
| Fibromyalgia | <1 | <1 | <1 | <1 | <1 | <1 | <1 |
| Injuries of nerves and spinal cord and paralytic syndromes | <1 | <1 | <1 | 2 | 2 | <1 | 2 |
| Blindness | <1 | <1 | <1 | <1 | 2 | <1 | 2 |
| Tinnitus | 1 | 2 | 2 | 4 | 3 | 2 | 1 |
| Congenital malformations, deformations and chromosomal abnormalities | <1 | 1 | 2 | 5 | 5 | 3 | 1 |
