# Supplementary material for: Patterns of multimorbidity and demographic profile of latent classes in a Danish population—A register-based study
Source: PLoS One. 2020 Aug 11;15(8):e0237375. doi: 10.1371/journal.pone.0237375 (PMC7418992; doi:10.1371/journal.pone.0237375)
Supplement: S5 Table — (DOCX) [file pone.0237375.s005.docx]

**Table S5: Fit statistics for analyses in the three age groups**

| **Number of latent classes** | **AIC** | **Change in AIC** | **BIC** | **Change in BIC** |
| --- | --- | --- | --- | --- |
| **65+ years** |  |  |  |  |
| **2** | 181685,1 |  | 182597,3 |  |
| **3** | 167613 | -14072 | 168986,1 | -13611 |
| **4** | 159110,8 | -8502 | 160944,7 | -8041 |
| **5** | 155250,7 | -3860 | 157545,6 | -3399 |
| **6** | 151649,2 | -3601 | 154405 | -3141 |
| **7** | 150198,8 | -1450 | 153415,5 | -990 |
| **8** | 148930,6 | -1268 | 152608,1 | -807 |
| **9** | 147395,2 | -1535 | 151533,7 | -1074 |
| **45-64 years** |  |  |  |  |
| **2** | 122584,9 |  | 123528,4 |  |
| **3** | 105794,8 | -16790 | 107214,9 | -16313 |
| **4** | 100261,9 | -5533 | 102158,7 | -5056 |
| **5** | 95982,05 | -4280 | 98355,51 | -3803 |
| **6** | 93169,98 | -2812 | 96020,11 | -2335 |
| **7** | 90989,45 | -2181 | 94316,26 | -1704 |
| **8** | 89285,95 | -1704 | 93089,44 | -1227 |
| **9** | 88230,62 | -1055 | 92510,79 | -579 |
| **16-44 years** |  |  |  |  |
| **2** | 50438,70 |  | 51412,77 |  |
| **3** | 43474,68 | -6964 | 44940,86 | -6472 |
| **4** | 38393,73 | -5081 | 40352,05 | -4589 |
| **5** | 35745,95 | -2648 | 38196,42 | -2156 |
| **6** | 34550,58 | -1195 | 37493,19 | -703 |
| **7** | 33827,22 | -723 | 37261,97 | -231 |
| **8** | 33251 | -576 | 37177,9 | -84 |
| **9** | 32681,7 | -569 | 37100,7 | -77 |

AIC: Akaike Information Criterion; BIC: Bayesian Information Criterion
