# Supplementary material for: Patterns of multimorbidity and demographic profile of latent classes in a Danish population—A register-based study
Source: PLoS One. 2020 Aug 11;15(8):e0237375. doi: 10.1371/journal.pone.0237375 (PMC7418992; doi:10.1371/journal.pone.0237375)
Supplement: S6 Table — (DOCX) [file pone.0237375.s006.docx]

**Table S6: Educational level of individuals by assigned classes in the age group 65+ years**

|  | **’No or few diseases’**  **46.3% §**  **(n=50,605)** | | **’Diabetes, cholesterol’**  **25.2%**  **(n=27,502)** | | **’Heart diseases’**  **8.3%**  **(n=9,065)** | | **’Back disease, asthma, allergy’**  **6.3%**  **(n=6,866)** | | **’Many diseases’**  **5.4%**  **(n=5,884)** | | **’COPD, cancer, liver disease’**  **4.4%**  **(n=4,756)** | | **’Mental illness, epilepsy’**  **4.2%**  **(n=4,640)** | | ***p*** |
| --- | --- | --- | --- | --- | --- | --- | --- | --- | --- | --- | --- | --- | --- | --- | --- |
|  | % | OR | % | OR [95%CI]^1^ | % | OR [95%CI]^1^ | % | OR [95%CI]^1^ | % | OR [95%CI]^1^ | % | OR [95%CI]^1^ | % | OR [95%CI]^1^ |  |
| **Educational level**  Missing  Elementary school  Short education^2^  Medium/long educ.^3^ § | 2.5  31.5  43.5  22.6 | 1.0  1.0  1.0  1.0 | 2.1  38.8  43.4  15.7 | 1.0 [0.9;1.1]  1.7 [1.6;1.8]  1.4 [1.4;1.5]  1.0 | 3.1  40.4  39.7  16.9 | 0.8 [0.7;0.9]  1.3 [1.3;1.4]  1.2 [1.1;1.2]  1.0 | 2.4  38.2  40.3  19.1 | 0.9 [0.8;1.1]  1.2 [1.1;1.3]  1.1 [1.0;1.2]  1.0 | 2.6  47.7  38.3  11.4 | 1.2 [1.0;1.5]  2.5 [2.2;2.7]  1.7 [1.5;1.8]  1.0 | 2.3  43.1  39.2  15.5 | 1.0 [0.8;1.2]  1.7 [1.5;1.8]  1.3 [1.2;1.4]  1.0 | 3.3  46.0  35.1  15.5 | 1.0 [0.9;1.3]  1.6 [1.5;1.8]  1.2 [1.1;1.3]  1.0 | *** |

***: p<0.001; §: reference group; OR: Odds ratio compared to the reference group of being in a multimorbidity class compared to the reference class; *p*: Chi^2^-test for univariate association between demographic variable and classes; ^1^Adjusted for age and sex. ^2^ Completed high school, vocational school, or short tertiary education. ^3^ Completed medium or long tertiary education (>3 years)
