# Supplementary material for: Patterns of multimorbidity and demographic profile of latent classes in a Danish population—A register-based study
Source: PLoS One. 2020 Aug 11;15(8):e0237375. doi: 10.1371/journal.pone.0237375 (PMC7418992; doi:10.1371/journal.pone.0237375)
Supplement: S7 Table — (DOCX) [file pone.0237375.s007.docx]

**Table S7: Educational level and employment status of individuals by assigned classes in the age group 45-64 years**

|  | **’No or few diseases’**  **69.4%** §  **(n=105,416)** | | | **’Diabetes, cholesterol’**  **11.4%**  **(n=17,349)** | | **’Bone-, joint diseases’**  **9.9%**  **(n=15,002)** | | | | **’Mental illness, epilepsy’**  **2.9%**  **(n=4,337)** | | | **’Heart diseases’**  **2.3%**  **(n=3,439)** | | | **’Many diseases’**  **2.1%**  **(n=3,201)** | | | **’Asthma, allergy’**  **2.1%**  **(n=3,126)** | | | ***p*** |  |
| --- | --- | --- | --- | --- | --- | --- | --- | --- | --- | --- | --- | --- | --- | --- | --- | --- | --- | --- | --- | --- | --- | --- | --- |
|  | % | OR | % | | OR  [95%CI]^1^ | | % | OR  [95%CI]^1^ | % | | OR  [95%CI]^1^ | % | | OR  [95%CI]^1^ | % | | OR  [95%CI]^1^ | % | | OR  [95%CI]^1^ |  | | |
| **Educational level**  Missing  Elementary school  Short education^2^  Medium/long educ.^3^ § | 3.5  18.4  49.7  28.5 | 1.0  1.0  1.0  1.0 | 2.8  27.6  50.6  19.0 | | 1.3 [1.2;1.4]  2.0 [1.9;2.1]  1.5 [1.4;1.6]  1.0 | | 2.4  26.2  47.8  23.7 | 1.0 [0.9;1.1]  1.7 [1.7;1.8]  1.3 [1.2;1.3]  1.0 | 4.4  32.6  42.2  20.9 | | 1.9 [1.6;2.2]  2.5 [2.3;2.8]  1.2 [1.1;1.3]  1.0 | 2.7  27.9  49.9  19.5 | | 1.2 [1.0;1.5]  2.0 [1.8;2.2]  1.4 [1.3;1.5]  1.0 | 3.6  39.9  42.5  14.0 | | 2.6 [2.1;3.2]  4.2 [3.8;4.7]  1.9 [1.7;2.1]  1.0 | 1.9  19.0  45.6  33.6 | | 0.5 [0.4;0.7]  0.9 [0.8;1.0]  0.9 [0.8;0.9]  1.0 | *** | | |
| **Employment status**  Working §  Unemployed  Sick leave etc.^4^  Early retirement pens.  Retired  Other | 82.9  4.9  0.6  5.3  3.0  3.3 | 1.0  1.0  1.0  1.0  1.0  1.0 | 66.7  7.5  1.1  14.5  7.7  2.6 | | 1.0  2.0 [1.9;2.2]  2.3 [2.0;2.8]  2.9 [2.7;3.0]  1.3 [1.2;1.4]  0.9 [0.8;0.9] | | 59.0  11.5  1.8  19.9  5.4  2.4 | 1.0  3.3 [3.1;3.5]  4.2 [3.6;4.8]  4.5 [4.3;4.8]  1.3 [1.2;1.5]  0.9 [0.8;1.0] | 27.4  22.7  2.8  42.9  1.8  2.4 | | 1.0  13.7 [12.6;15.0]  14.1 [11.5;17.3]  24.4 [22.6;26.4]  2.0 [1.6;2.5]  2.1 [1.7;2.6] | 61.3  9.0  1.4  17.5  8.0  2.9 | | 1.0  2.7 [2.4;3.1]  3.2 [2.4;4.4]  3.7 [3.4;4.1]  1.3 [1.2;1.5]  1.0 [0.8;1.2] | 20.5  17.2  2.6  53.0  4.5  2.1 | | 1.0  15.0 [13.3;16.9]  17.5 [13.7;22.3]  33.3 [30.3;36.7]  2.5 [2.1;3.0]  2.2 [1.7;2.9] | 73.5  8.1  0.9  12.5  3.0  2.0 | | 1.0  1.8 [1.6;2.1]  1.7 [1.2;2.5]  2.5 [2.2;2.8]  0.9 [0.8;1.2]  0.6 [0.5;0.8] | *** | | |

***: p<0.001; §: reference group; OR: Odds ratio compared to the reference group of being in a multimorbidity class compared to the reference class; *p*: Chi^2^-test for univariate association between demographic variable and classes; ^1^Adjusted for age and sex. ^2^ Completed high school, vocational school, or short tertiary education. ^3^ Completed medium or long tertiary education (>3 years). ^4^ Includes individuals on sick leave, maternity leave, or other types of leave related to for example training
