# Supplementary material for: Patterns of multimorbidity and demographic profile of latent classes in a Danish population—A register-based study
Source: PLoS One. 2020 Aug 11;15(8):e0237375. doi: 10.1371/journal.pone.0237375 (PMC7418992; doi:10.1371/journal.pone.0237375)
Supplement: S8 Table — (DOCX) [file pone.0237375.s008.docx]

**Table S8: Educational level and employment status of individuals by assigned classes in the age group 16-44 years**

|  | **’No or few diseases’**  **90.3%** §  **(n=189,202)** | | **’Bone-, joint diseases’**  **3.8%**  **(n=7,962)** | | **’Mental illness, epilepsy’**  **3.5%**  **(n=7,378)** | | **’Asthma, allergy’**  **1.4%**  **(n=2,985)** | | **’Diabetes, heart diseases’**  **1.0%**  **(n=2,079)** | | ***p*** |
| --- | --- | --- | --- | --- | --- | --- | --- | --- | --- | --- | --- |
|  | n (%) | OR | n (%) | OR [95%CI]^1^ | n (%) | OR [95%CI]^1^ | n (%) | OR [95%CI]^1^ | n (%) | OR [95%CI]^1^ |  |
| **Educational level**  Missing  Elementary school  Short education^2^  Medium/long educ.^3^ § | 10.1  28.8  38.9  22.2 | 1.0  1.0  1.0  1.0 | 3.4  29.0  44.0  23.7 | 0.5 [0.4;0.5]  2.2 [2.1;2.4]  1.4 [1.3;1.5]  1.0 | 4.7  44.2  36.2  14.9 | 0.9 [0.8;1.1]  4.5 [4.2;4.9]  1.8 [1.7;1.9]  1.0 | 2.6  31.3  39.6  26.5 | 0.2 [0.2;0.3]  1.0 [0.9;1.2]  0.9 [0.8;1.0]  1.0 | 5.6  30.8  46.1  17.5 | 1.2 [1.0;1.5]  4.0 [3.5;4.6]  1.9 [1.7;2.2]  1.0 | *** |
| **Employment status**  Working §  Unemployed  Sick leave etc.^4^  Early retirement pens.  Other  Student | 59.3  5.6  1.3  1.3  10.0  22.5 | 1.0  1.0  1.0  1.0  1.0  1.0 | 56.8  16.0  2.8  8.1  4.7  11.7 | 1.0  3.1 [2.9;3.3]  2.2 [1.9;2.5]  6.3 [5.8;6.9  0.8 [0.7;0.9]  1.0 [1.0;1.1] | 28.4  32.2  3.7  13.2  7.6  14.9 | 1.0  11.9 [11.2;12.6]  5.3 [4.7;6.1]  21.4 [19.6;23.3]  1.9 [1.7;2.1]  1.7 [1.6;1.9] | 57.7  6.0  1.7  1.8  7.1  25.7 | 1.0  1.1 [0.9;1.3]  1.2 [0.9;1.7]  1.5 [1.1;1.9]  0.8 [0.7;0.9]  1.3 [1.2;1.4] | 57.6  18.2  2.0  14.6  3.5  4.1 | 1.0  4.0 [3.6;4.5]  2.4 [1.8;3.3]  9.9 [8.6;11.3]  1.1 [0.8;1.3]  1.1 [0.9;1.5] | *** |

***: p<0.001; §: reference group; OR: Odds ratio compared to the reference group of being in a multimorbidity class compared to the reference class; *p*: Chi^2^-test for univariate association between demographic variable and classes; ^1^Adjusted for age and sex. ^2^ Completed high school, vocational school, or short tertiary education. ^3^ Completed medium or long tertiary education (>3 years). ^4^ Includes individuals on sick leave, maternity leave, or other types of leave related to for example training
