# Supplementary material for: Patterns of multimorbidity and demographic profile of latent classes in a Danish population—A register-based study
Source: PLoS One. 2020 Aug 11;15(8):e0237375. doi: 10.1371/journal.pone.0237375 (PMC7418992; doi:10.1371/journal.pone.0237375)
Supplement: S9 Table — Results weighted for probability of class membership. (DOCX) [file pone.0237375.s009.docx]

**Table S9: Demographic profile of individuals by assigned classes in the age group 45-64 years. Results weighted for probability of class membership.**

|  | **’No or few diseases’**  **69.4%** §  **(n=105,416)** | | | **’Diabetes, cholesterol’**  **11.4%**  **(n=17,349)** | | **’Bone-, joint diseases’**  **9.9%**  **(n=15,002)** | | | | **’Mental illness, epilepsy’**  **2.9%**  **(n=4,337)** | | | **’Heart diseases’**  **2.3%**  **(n=3,439)** | | | **’Many diseases’**  **2.1%**  **(n=3,201)** | | | **’Asthma, allergy’**  **2.1%**  **(n=3,126)** | | | ***p*** |  |
| --- | --- | --- | --- | --- | --- | --- | --- | --- | --- | --- | --- | --- | --- | --- | --- | --- | --- | --- | --- | --- | --- | --- | --- |
|  | % | OR | % | | OR  [95%CI]^1^ | | % | OR  [95%CI]^1^ | % | | OR  [95%CI]^1^ | % | | OR  [95%CI]^1^ | % | | OR  [95%CI]^1^ | % | | OR  [95%CI]^1^ |  | | |
| **Age**  45-54 §  55-64 | 59.7  40.3 | 1.0  1.0 | 33.7  66.3 | | 1.0  2.9 [2.8;3.0] | | 48.6  51.4 | 1.0  1.6 [1.5;1.6] | 57.4  42.6 | | 1.0  1.1 [1.0;1.1] | 29.8  70.2 | | 1.0  3.5 [3.3;3.7] | 32.8  67.2 | | 1.0  3.0 [2.8;3.2] | 56.3  43.7 | | 1.0  1.1 [1.1;1.2] | *** | | |
| **Sex**  Men §  Women | 51.6  48.4 | 1.0  1.0 | 58.3  41.7 | | 1.0  0.7 [0.7;0.7] | | 41.2  58.8 | 1.0  1.9 [1.9;2.0] | 42.5  57.5 | | 1.0  1.4 [1.3;1.5] | 63.0  37.0 | | 1.0  0.5 [0.5;0.6] | 44.8  55.2 | | 1.0  1.3 [1.3;1.4] | 38.6  61.4 | | 1.0  2.0 [1.9;2.2] | *** | | |
| **Country of origin**  Danish §  Other Western  Non-Western | 90.3  4.0  5.7 | 1.0  1.0  1.0 | 88.8  2.4  8.8 | | 1.0  0.7 [0.6;0.7]  1.8 [1.7;2.0] | | 90.9  2.9  6.2 | 1.0  0.8 [0.7;0.8]  1.1 [1.1;1.2] | 86.1  3.0  10.9 | | 1.0  0.8 [0.7;0.9]  2.0 [1.9;2.2] | 91.7  2.4  5.9 | | 1.0  0.6 [0.5;0.8]  1.2 [1.1;1.4] | 87.9  2.5  9.6 | | 1.0  0.7 [0.6;0.8]  2.1 [1.8;2.3] | 90.3  2.8  6.9 | | 1.0  0.7 [0.6;0.9]  1.2 [1.1;1.4] | *** | | |
| **Marital status**  Unmarried  Married § | 38.0  62.0 | 1.0  1.0 | 37.1  62.9 | | 1.0 [1.0;1.1]  1.0 | | 39.7  60.3 | 1.1 [1.1;1.1]  1.0 | 55.2  44.8 | | 2.0 [1.9;2.1]  1.0 | 39.6  60.4 | | 1.2 [1.1;1.2]  1.0 | 50.2  49.8 | | 1.8 [1.7;1.9]  1.0 | 36.7  63.3 | | 1.0 [0.9;1.0]  1.0 | *** | | |

***: p<0.001; §: reference group; OR: Odds ratio compared to the reference group of being in a multimorbidity class compared to the reference class; *p*: Chi^2^-test for univariate association between demographic variable and classes; ^1^Adjusted for age and sex
