# Supplementary material for: Patterns of multimorbidity and demographic profile of latent classes in a Danish population—A register-based study
Source: PLoS One. 2020 Aug 11;15(8):e0237375. doi: 10.1371/journal.pone.0237375 (PMC7418992; doi:10.1371/journal.pone.0237375)
Supplement: Attachment — Submitted filename: Response to reviewers.docx [file pone.0237375.s010.docx]

Response to reviewers

We wish to thank the reviewers for taking the time to review the manuscript and for their comments and suggestions, which we have addressed individually below.

|  | Review comments | Author response |
| --- | --- | --- |
| REVIEWER NO. 1 | | |
| 1 | Reviewer #1: General remark: The aim of this study is to identify and describe demographic characteristics of multimorbidity classes in three age groups by applying latent class analysis on registry data from a random sample of the Danish population. I find the study interesting and relevant, but there are a number of methodological weaknesses in the study that I think should be addressed. | We thank the reviewer for this positive appraisal of the relevance of the study. |
| 2 | Page 1, line 1 I suggest that the authors consider replacing 'pattern' with 'patterns'. | We have changed the title as suggested. |
| 3 | Page 3, line 50-53 This section is difficult to understand. While knowledge about LCA is still limited among many epidemiologists and other health researcher, I suggest that the authors use a more straightforward and understandable explanation of LCA. See, e.g. Mariano Porcu, Francesca Giambona (2017). Introduction to Latent Class Analysis With Applications. The Journal of Early Adolescence, 37, 1: 129-158, or some of the standard literature on LCA (e.g. Bartholomew, Knott, & Moustaki (2011) or McCutcheon (1987)). | We have revised the sentence and included Porcu & Giambona among the references:  “One method previously used is latent class analysis (LCA) which is used to study phenomena that cannot be directly measured. With LCA it is possible to divide a population into categories of an unobserved variable {Porcu, 2017 #678}. If the LCA is based on information on diseases this will result in categories that represent groups of individuals with different disease profiles.” *(Background, paragraph 2)* |
| 4 | Page 3, line 54-55 'This technique has been used in previous studies identifying patterns of multimorbidity in different populations (6-8).' The authors should add this study to the reference list:  Park B, Lee HA, Park H. Use of latent class analysis to identify multimorbidity patterns and associated factors in Korean adults aged 50 years and older. PLoS One. 019;14(11):e0216259. Published 2019 Nov 13. doi:10.1371/journal.pone.0216259 | The study has been added to refence list. |
| 5 | Page 4, line 75 Which register was the sample extracted from? | The sample was drawn from the Danish Civil Registration System. This information has been added to the manuscript:  “This was drawn from register the Danish Civil Registration System which includes all Danish residents” *(Methods, paragraph 1)* |
| 6 | Page 5, line 107 'LCA was carried out separately for three age groups…' A major disadvantage of making three instead of one latent class model is that one cannot compare the prevalence of latent classes across age because the classes are specific to the age groups.  In my opinion, it is a better analytical strategy to estimate a latent class model for the entire sample and either include age in the model at par with the other indicators or, even better, include age as a covariate (cf. Dayton, C. M., & Macready, G. B. (1988). Concomitant variable latent class models. Journal of the American Statistical Association, 83, 173-178). The latter type of model allows you to answer the question: Is age predictive of class membership? A third way to proceed with this is to use a so-called three-step procedure, cf. below. | We chose to separate the population into three age groups, because we expected that the disease burden would differ substantially between the age groups, and that multimorbidity would also be expressed differently. Also, we expected that an LCA performed on the entire population would be heavily influenced by the disease profile of the elderly population, and we feared that the pattern of multimorbidity in the younger age groups would be impossible to deduce from these findings. |
| 7 | Page 6, line 115-117 'To asses associations between the demographic variables and the different classes, we applied a multinomial logistic regression model, which was adjusted for age and sex.' Here a three-step procedure is utilized within each of the three LCA models.  This involves (1) estimating a LCA model, using only data on the indicators of the latent classes, (2) assigning a latent class to each individual based on the model from step 1, and (3) estimating a logistic regression model with the assigned values from step 2 as dependent variable.  The three-step method has the flaw that the values assigned in its second step are not equal to the true values of the latent classes as defined by the first step (the assignment to latent classes is probabilistic). This creates a measurement error (misclassification) problem which means that the third step will yield biased estimates of the regression model.  Several methods have been developed to correct for this bias (cf. Bakk, Zs., Tekle, F.B., and Vermunt, J.K. (2013). Estimating the association between latent class membership and external variables using bias adjusted three-step approaches. Sociological Methodology, 43, 272-311). My suggestion to the authors is that they apply one of these bias-adjusted methods. | Analyses using the described methods to adjust for bias did as expected result in larger estimates. For the bigger classes, the differences were modest, but for the smaller classes, the approach resulted in somewhat larger estimates. Based on this finding, we have added the following paragraph to the discussion:  “A third limitation is that the method of assigning individuals to the class with the highest probability could result in biased estimates in the multinomial logistic regression analyses. This bias seemed to influence the estimates for class membership in the smaller classes, where the presented results would be conservative estimates of associations.” *(Discussion, paragraph 8)* |
| 8 | Page 7, table 1 How is 'Short education' and 'Medium/long education' defined? | The explanation has been added as a footnote to table 1:  “Short education: Completed high school, vocational school, or short tertiary education; Medium/long education: Completed medium or long tertiary education (> 3 years)” |
| 9 | Page 8, line 133-143 When conducting an LCA, a major decision in the process of model specification is choosing the number of latent classes to retain. On page 5, lines 103-106, the six criteria that were used to select the number of latent classes are mentioned. In order to allow the reader to assess the quality of the model chosen, I suggest that the authors in this section briefly describe how the six criteria have been applied and weighed against each other. How well does the selected model fit the data according to the criteria? How good is the latent class separation? Etc. The authors might consider adding a supplementary table with more detailed information. | The requested table has been added to the supplements (table S5), and the following description has been added to the methods:  “Nine was the maximum number of clusters evaluated. The AIC and BIC values continued to decrease as more latent classes were added to the model, but models with distinguishable classes were prioritised.” *(Methods, Statistical analyses)* |
| 10 | Page 14, line 22 'Overall, the classes were similar in the three age groups, but varied in size…' I agree with this finding, which shows that nothing is really gained by using three models rather than one. | We believe that the finding of relatively similar patterns between age groups is an important finding in itself, and not a finding that we would have been able to observe, if we had used a single model. |
| REVIEWER NO. 2 | | |
| 11 | With the ageing of the population, multimorbidity is an important public health problem and epidemiological data on this topic, is therefore highly relevant. Even though other studies have investigated the pattern of multimorbidity, this paper is interesting and adds useful information to the existing literature. The study is based on a large random sample of Danish population. The use of register-based information and separate age-groups analyses are other strengths of the study. The authors used appropriate methods to explore the research questions. I only have a few minor remarks. | We thank the reviewer for this comment and of course agree on the importance and relevance of this topic. |
| 12 | Line 43-44 : The authors state that multimorbidity is a public health burden today and is estimated to increase in the future. There is lack of data to support this statement. It will be useful to provide some information, e.g., prevalence of multimorbidity worldwide and/or in Denmark. | We have not included an estimate of the prevalence of multimorbidity as this varies greatly with study population, methods to estimate multimorbidity, definition of multimorbidity, types of diseases, and definition of diseases. We have instead edited the following paragraph in the background:  “Studies on multimorbidity vary substantially in study population and the applied methods to estimate multimorbidity, as well as the number of diseases, types of diseases, and definition of diseases included. For this reason, reported estimates of prevalence of multimorbidity vary greatly in the literature” *(Background, paragraph 2)* |
| 13 | The sentence “Apply methods to assess multimorbidity include…..” should be moved in the method section. Furthermore, authors should justify why LCA has been chosen instead of other methods. | The sentence has been moved to the section on statistical analyses in the methods. We have also included the following description of the justification for this method:  “In this study, LCA was used to assess multimorbidity as this approach is exploratory and does not entail a priori assumptions about patterns of multimorbidity. Also, in LCA the latent variable is considered to be a discrete variable as opposed to a continuous variable. However, we do not consider the LCA approach to be superior to other methods.” *(Methods, Statistical analyses)* |
| 14 | Please clarify how the 47 chronic diseases were clinically validated. Those the diseases selected based on their ICD-10 codes or others? | The diseases were based on information on ICD-10 codes from the National Patient Registry and the National Psychiatry Central Research Register and for some diseases also on information on ATC codes and indication codes from the National Prescription Registry.  The validity is thus high for diseases which usually result in hospital contacts, but may be lower for diseases that do not cause hospital contacts. We have added a sentence on the lower validity of prescription information in the following paragraph of the discussion:  “The limitations of the study include that diseases are not included if they have only been treated in primary care and no disease specific treatment drugs have been prescribed. Therefore, diseases such as many mental illnesses, allergy and musculoskeletal diseases may be under reported. However, we tried to take this into account by including information on prescriptions which should allow for at least partly detection of some of these diseases, but the validity of this information may be lower than that of ICD-10 codes.” *(Discussion, paragraph 8)* |
| 15 | Line 84 - 86, it is mentioned that “Identification of diseases was made by applying diagnoses and purchase of prescribed medications primarily based on algorithms applied in previous studies…”. In my opinion, it is important to provide more detailed on those algorithms in order to facilitate readers comprehension. An overview of chronic diseases is presented in additional table S1 and it is mentioned that the algorithms will also find in the same table. However, I miss the later in table S1, please address this. | Information on the included ICD-codes and prescription medicines was regrettably not included in Table S1 but has now been added to the table. Thank you for bringing this to our attention. |
| 16 | Referring to my previous remark, information on the methods used to identify the pattern of multimorbidity should be address in this section. Furthermore, please motivate the use of LCA compared to other methods. | See reply to comment #13. |
| 17 | A large number of sociodemographic covariates are included in the study. However, I find it weird that a univariate logistic regression model is used to asses associations between the demographic variables and the different classes instead of multivariate one. Why? This may affect interpretation of the results. | The results in table 3-5 and figure 1-2 have all been adjusted for age and sex. The remaining variables are not mutually adjusted as we do not wish to infer causal associations but only characterise the multimorbidity groups. Also, as the temporal relation between many of the variables is somewhat difficult to entangle we also fear that adjustments may in many cases be overadjustments with mediators included as confounders. |
| 18 | As one of the strength of the present study in contrast to previous studies, is the identification of age specific patterns of multimorbidity, it would be useful to provide in the table 2 the mean number of diseases by age-group, not only by classes of diseases. Such information could also point out in what extend multimorbidity can differ across age-groups and why age-specific analyses are useful. | The mean no. of diseases is 0.5 in the youngest age group, 1.4 in the middle age group, and 2.8 in the oldest age group.  This information has now been added to table 2. |
| 19 | Compared to other studies, authors found a large range of classes. How they can justify this finding? | We believe that the relatively large number of classes is mainly caused by the  large sample size of more than 470,000 individuals. However, the Danish study by Larsen et al. mentioned below also found seven classes in their LCA. |
| 20 | How those the authors interpret the fact that they found similar classes across age-group? Do this meant youngest and older people suffer from the same chronic diseases? problem of misclassification? | The relatively similar classes across age groups is possibly a consequence of the LCA being heavily influenced by the most prevalent diseases in each age group. Though the disease prevalences in the younger age groups are naturally lower, it seems that there are not very large discrepancies in *which* diseases are the most prevalent diseases between the age groups. |
| 21 | In the conclusion, a summary of the results is presented and ideas for future researches. I miss implications of the study for public health and health policymakers. | We have added the following sentence to the conclusion:  “The findings also highlight that healthcare systems need to be able to accommodate complex disease pictures among patients and that these may be associated with social circumstances which should also be taken into account in the treatment of these patients.” *(Discussion, paragraph 9)* |
| 22 | An important study has been carried out in Denmark in the same area which is lacking in this article. Authors should consider adding this reference at least in the introduction and or discussion:  Breinholt Larsen F, Hauge Pedersen M, Friis K, Glümer C, Lasgaard M. Patterns of Multimorbidity in the General Danish Population. A Latent Class Analysis. European Journal of Public Health. 2018 Nov 1;28(suppl_4):cky214-062. | The mentioned publication refers to a conference presentation, which we did not include in the references but the findings are described in the reference:  Larsen FB, Pedersen MH, Friis K, Glumer C, Lasgaard M. A Latent Class Analysis of Multimorbidity and the Relationship to Socio-Demographic Factors and Health-Related Quality of Life. A National Population-Based Study of 162,283 Danish Adults. PloS one. 2017;12(1):e0169426  which in the manuscript is referred to as reference no. 7 |
| REVIEWER NO. 3 | | |
| 23 | Reviewer #3: The authors have conducted a sound analyses of a large sample of high quality Danish data (~470,000 individuals) in order to characterize detailed multimorbidity profiles across a sample of the population. Determining multi morbidity disease profiles and the underlying demographics which may be associated with those profiles is of interest to the medical community and is very topical. The link with educational data is strong and unique - as not many other countries are able to link such data to healthcare information at this sacale. Whilst the current paper uses appropriate and advanced latent class analyses techniques to define multi morbidity clusters from 47 individual diseases - the main contribution of the study is largely descriptive as there are no analyses of the outcomes associated with different MM clusters. Without information on the impact of these newly define MM clusters it is not clear what the clinical impact or recommendation for changes to healthcare is on the basis of this research. Whilst it is interesting to see that females are more at risk of certain MM presentations compared to males for example - the potential for targetted intervention for prevention of these MM presentations in females will only be made if we know whether these MM clusters are particularly important in terms of quality of life or mortality burden for example. The Academy of Medical Sciences report on multimorbidity clearly highlights the lack of studies looking at MM and association with outcomes as a key problem and knowledge gap. This limitation should be made clear - if not able to be addressed with the current data. | We thank the reviewer for the positive appraisal of the study.  We have included the following sentence addressing this limitation in the discussion:  “Finally, this study does not include information on associations between multimorbidity and possible consequences such as hospital admissions, employment, and mortality, which could help inform intervention strategies.” *(Discussion, paragraph 8)* |
| 24 | 1. Missing key references in the introduction for work which has used LCA to assess MM clusters in detail - including a number of references found in the AMS report, particularly the work by Hall et al 2018 in PloS Medicine using LCA to look at clusters of multimorbidity and long term outcomes. | We have included the following paragraph in the background on studies (Larsen et al.; Wang et al.; Hall et al.) describing associations between multimorbidity and health outcomes:  “Some studies have also shown that pattern of multimorbidity is associated with outcomes such as mortality and health-related quality of life.” *(Background, paragraph 4)* |
| 25 | 2. The authors claim the risk of selection bias in the study is limited because this is a registry based analyses. However - the data included represents a 10% sample of the population - without clear and detailed inforamtion alongside table 1 of the characteristics of the full population it is impossible to say whether or not these 10% are indeed representative. More information on this in needed to support the claim. | The 10 % random sample from the Danish population was drawn by Statistics Denmark from the Danish Civil Registration System, which includes all residents in Denmark, and therefore there is no selection bias in the sampling.  Compared to the population data the deviations in 10-year age *sex groups were less than 1% up to the 70-79 years group, less than 3% up to the 90-99 years group, and less than 5% for the age group 100-109 years.  We have added the following sentence to the methods:  “This sample was drawn from the Danish Civil Registration System which includes all Danish residents and the deviation in age/sex distribution of the sample from the total population using 10 years age group was less than 1% up to 79 years” *(Methods, paragraph 1)* |
| 26 | 3. What is the justification / reasoning for the three specific age groups chosen? Are these based on particular clinically relevant age cut offs/made around healthcare service provision? please make clear throughout. | We have added a description of the reasoning behind the specific age groups:  “These age groups were defined as 1) a young age group based on individuals who would be expected to have no or few diseases (16-44 years); 2) a middle-aged group based on individuals who would to some degree be expected to be disease free (45-64 years); and 3), an elderly group based on individuals who have mainly retired from the work force and would be expected to be affected by one or more diseases (65+ years).” *(Methods, paragraph 1)* |
| 27 | 4. I could not find any information about the choices for the specific clusters - the authors state in the methods this was made based on model fit statistics as well as how meaningful the emerging classes were - however provide no tables of model fit statistics and discussion of class cut offs. This is important information to include - to ensure scientific rigour and transparency in the decision making process. Please also clarify the maximum number of clusters that were checked. | The requested information has been included in supplement table S5 and the following sentences have been added to the Methods:  “Nine was the maximum number of clusters evaluated. The AIC and BIC values continued to decrease as more latent classes were added to the model, but models with distinguishable classes were prioritised.” *(Methods, Statistical analyses)* |
| 28 | 5. Disease definitions - more detailed is required on which conditions, or how many individuals, were identified as having a particular disease through the prescription of medications only. Was this done only for certain prescriptions where the medication would only ever be prescribed for that particular disease ? Many drugs will be used to treat a number of conditions, therefore I question the accuracy of a 'diagnoses' being inferred based on prescription of one medication twice. A sensitivity analyses of classes and disease profiles which excludes the prescription only 'diagnoses' is strongly advised so that the impact of this decision on the final outcomes can be determined. | Regrettably, supplement table S1 in our first submission did not include information on ICD-10 codes, ATC codes and indication codes used for identification of the individual diseases. This has now been included.  It is true that the use of prescription information is a challenge due to overlapping use of medications for different diseases.  We have tried to accommodate for this by including indication codes where relevant. These hold information on the underlying disease treated. The indication codes have been used to distinguish between especially psychiatric diseases with overlapping medications used. Our algorithm for identification ensures that no diseases have overlapping definitions (see table S1), and we hope that this information will help convince the reviewer of the accuracy of the diagnoses. |
| 29 | 6. The paper needs more detailed information with regards to the make up of the clusters - this is provided in tables in the supplement, but needs to be presented up front in the main paper as without it the over arching cluster descriptions can't be easily interpreted. Ideally - the authors should try to determine the most accessible way to present this high level of detailed information visually or graphically rather than in tabular format. | We have added a figure (Figure 1) to the manuscript, which shows the prevalence of the most prevalent diseases and of the diseases with the largest variations in prevalence between classes. |
| 30 | 7. Please ensure all tables are fully annotated with additional inforamtion in footnotes. In table 1 - please clarify the full definition of "sick leave etc.". Also in Table 1 please add a footnote to say what the '-' reefers to - i.e. is this missing data or not applicable (if the latter - why is it not applicable for a over 65 year old to be on sick leave?). For marital status - please be more specific. Current/ever/at baseline?). | We have added the following footnotes to table 1:  “^1^ Completed high school, vocational school, or short tertiary education  ^2^ Completed medium or long tertiary education (> 3 years)  ^3^ Includes individuals on sick leave, maternity leave, or other types of leave related to for example training  ^4^ Marital status in the year 2017” |
