# Supplementary material for: Patterns of multimorbidity and demographic profile of latent classes in a Danish population—A register-based study
Source: PLoS One. 2020 Aug 11;15(8):e0237375. doi: 10.1371/journal.pone.0237375 (PMC7418992; doi:10.1371/journal.pone.0237375)
Supplement: Attachment — Submitted filename: Rebuttal letter July.docx [file pone.0237375.s011.docx]

Response to reviewers

We thank the reviewer for reviewing the manuscript again, and we have addressed the raised concerns below.

Reviewer #1: Line 96-99: "A third limitation is that the method of assigning individuals to the class with the highest probability could result in biased estimates in the multinomial logistic regression analyses. This bias seemed to influence the estimates for class membership in the smaller classes, where the presented results would be conservative estimates of associations."

It is not possible for the reader to assess how the authors reached this conclusion. Have they done further analysis with another method? If so, which one? And if so, why didn't they report these results instead? I reiterate my recommendation from the first review to use a bias-adjusted method to investigate the association between multimorbidity classes and sociodemographic characteristics.

Reply:

We have now included results from a sensitivity analysis in the age group 45-64 years weighting the results for the probability of belonging to the class. These results were similar to the main results, and we have added the following descriptions to the manuscript along with a supplement table showing the results (S9):

“Bias may be introduced from assigning individuals to the class with the highest probability, and therefore a sensitivity analysis was conducted in which the multinomial logistic regression included weights based on the probability of belonging to this class. This analysis was based on data from the age group 45-64 years.” (Methods, Statistical analysis, p. 6)

“Sensitivity analyses weighted for the probability of belonging to a class in the age group 45-64 years showed similar results (table S9).” (Results, p. 11)

“The sensitivity analysis applying weights for the probability of belonging to the assigned class showed only small differences in results between the two groups, but it is possible that this method does not adequately address bias introduced in the analysis.” (Discussion, p. 18)

General comment: I still find this to be a worthwhile study. But the information it provides is very complex. No less than 19 latent patterns of disease have been identified within three age groups. The authors have gone a long way in communicating the results in graphs and tables, but most readers will have difficulty grasping the significance of the individual disease classes. Consider if this could be made clearer.

Reply:

We agree that the results are quite comprehensive. However, we believe the results answer our study aim. Also, one of our main objectives is to illustrate similarities and differences in classes across age groups which inherently results in many classes. We have done our best to make illustrations that makes it easier for the reader to grasp the results, but we are very open for other suggestions of alternative illustrations that the reviewer might have.
